# DeFiTrustChain: A DeFi-Enabled NFT and Escrow Framework for Secure Automotive Supply Chains in Smart Cities

**DOI:** 10.3390/s26010315

**Published:** 2026-01-03

**Authors:** Archana Kurde, Sushil Kumar Singh, Aziz Alotaibi

**Affiliations:** 1 Department of Computer Engineering, Marwadi University, Rajkot 360003, India; archana.kurde123777@marwadiuniversity.ac.in; 2 Department of Computer Science, College of Computers and Information Technology, Taif University, Taif 21944, Saudi Arabia

**Keywords:** decentralized finance (DeFi), tokenized asset management, conditional escrow protocol, permissioned blockchain

## Abstract

The rising usage of IoT devices in everyday life has formed smart cities that require the adoption of decentralized systems for a secure and transparent mechanism to manage asset exchange across automotive supply chains. Several existing Blockchain-based models built on public chains focus on traceability while overlooking scalability limits, transaction fees, conditional payment trust, or real-time delivery validation. We introduce DeFiTrustChain, a DeFi-enabled framework that combines free NFTs, escrow-based automation, and IoT verification within a Hyperledger Fabric network. It represents each vehicle using a unique NFT to capture the details of manufacturing and ownership, along with immutable asset verification. The payment release between stakeholders is governed by a dedicated escrow contract responsible for IoT-based delivery confirmation. The proposed framework ensures authenticated access and prevents identity misuse through integration of the Fabric Certificate Authority. The experimental results demonstrate the coherent and dependable execution of NFT creation, escrow enforcement, and IoT-triggered validation, with low local transaction processing time and consistent behavior across peers.

## 1. Introduction

The rapid proliferation of IoT devices being deployed worldwide is driving the emergence of increasingly connected and intelligent smart cities. These heterogeneous interconnected technologies give rise to the need for data-driven decision-making and efficient resource utilization. Heterogeneous subsystems such as smart buildings, healthcare, transportation, and supply chains are integrated to form a smart city to improve sustainability and quality of life. The infusion of these technologies introduces new challenges related to data privacy, scalability, interoperability among heterogeneous systems, and trust management. These challenges need to be addressed to attain the intended societal and economic benefits.

In the past decade, Blockchain technology has evolved enough to provide promising solutions for transparency, traceability, and decentralization. The integration of Blockchain with IoT-driven smart city applications can strengthen data integrity and introduce decentralized authority. The representation and secure management of digital assets are well supported by the permissioned Blockchain framework, Hyperledger Fabric, encompassing NFTs and smart contracts. These technologies can ensure product authenticity, automate conditional payments, and enable trustless transactions within smart supply chains. This research work showcases the implementation of a Blockchain-enabled IoT system for a smart automotive supply chain, aiming to enhance transparency, trust, and workflow efficiency among stakeholders within the network.

Despite the notable progress achieved by smart cities and IoT-based infrastructures, several challenges remarkably hinder the effective implementation of complex and data-sensitive environments, such as the supply chain. Traditional supply chains are managed by centralized authorities for data storage, verification, and handling of transactions. This leads to inefficiencies, low transparency, and vulnerability to data manipulation or single points of failure. The involvement of multiple stakeholders, such as manufacturers, dealers, and end customers, requires the system to be trustworthy and accountable across the network.

The recent advancements in Blockchain make it a promising tool for enhancing transparency, trust among stakeholders, and traceability within supply chains. Most existing Blockchain-based solutions rely on public Blockchain platforms that lack the scalability much needed for industrial deployment and privacy control. The permissioned environment, like Hyperledger Fabric, capable of representing unique physical assets such as vehicles through Non-Fungible Tokens (NFTs), is yet to be explored. Additionally, automation through smart contracts and escrow-enabled conditional payment has yet to be fully leveraged in this domain. These research gaps emphasize the need for a comprehensive framework to attain secure, transparent, and efficient supply chain operations. Within the proposed framework, NFTs are operated in a permissioned Blockchain space in which the creation and transfer of NFTs do not have to pay a gas-based transaction cost, unlike the public Blockchain systems such as Ethereum and associated networks, in which the price of a gas operation is variable, thus making the operation of NFTs more predictable and suitable to enterprise supply chain operations.

Decentralized Finance (DeFi) concepts have been applied in recent years in support of automated and conditional financial functions using smart contracts that are not restricted to public cryptocurrency ecosystems. In enterprise applications, escrow-based payment settlement (where money is locked away and automatically unlocked on meeting predetermined conditions) is more frequently being implemented as a DeFi-inspired mechanism instead of as a result of speculative trading or open financial markets. Automation of such conditional payment is especially applicable in the automotive supply chains, where the financial settlement is intertwined with the asset authentication, delivery verification, and compliance validation.

The primary objective of this study is to design and implement a transparent and traceable, Blockchain-enabled IoT framework for the smart automotive supply chain. Along with enhanced trust among all participating entities, this assures conditional payment. The proposed framework leverages Hyperledger Fabric as a permissioned Blockchain platform that utilizes Non-Fungible Tokens (NFTs) for the unique digital asset representation of a car. The framework automates conditional transactions by integrating smart contracts and escrow mechanisms that ensure transfer and payment only when predefined conditions are met. This approach provides transparency to the entire process of asset transfer, beginning from asset creation till its ownership transfer to the end customer. This approach minimizes human intervention, on top of mitigating the risk of fraud and disputes among stakeholders. This work presents a complete reference implementation, designed to validate the end-to-end operational workflow of the proposed architecture.

The proposed framework yields the following key contributions while pursuing the approach described:

- A unique digital asset identity using Non-Fungible Tokens (NFTs) and managing automotive assets across multiple stakeholders on the permissioned chain within the Hyperledger Fabric.
- Transparent and condition-driven financial settlements between manufacturers, dealers, and end users through an automated escrow-based payment system using smart contracts.
- Improved reliability and responsiveness of the supply chain network by integrating IoT data streams for real-time tracking and validation of asset status.
- Evaluation of latency and throughput under varying workloads for performance analysis and monitoring of the proposed framework using Prometheus and Grafana.
- Demonstration of the enhancement of the workflow efficiency, trust, and sustainability of next-generation automotive supply chains by integrating Blockchain and IoT through a comprehensive assessment of system feasibility.

### Related Work

This research provides a robust foundation for developing future-ready, decentralized smart supply chain systems by addressing critical gaps in asset authentication, payment automation, and trust management. The proposed model contributes to the broader goal of achieving sustainable and transparent digital ecosystems with the vision of intelligent and connected cities. The integration of Blockchain and IoT has the potential to enhance automation, traceability, and digital authorization in distributed environments that consist of heterogeneous users connected in the network from diverse locations. Alsadi et al. [1] proposed TruCert—a Blockchain-enabled product certification system for the automotive sector that ensures provenance verification but does not consider real-time IoT connectivity and asset tokenization. Chen et al. [2] proposed a Hyperledger Fabric–based traceability system with off-chain IPFS storage capable of managing efficiently large datasets for agricultural products. Iftekhar et al. [3] proposed a tamper-proof data storage for the safety of food through Blockchain over public Ethereum, although it has increased latency and reduced privacy control. Pajooh et al. [4] proposed an edge architecture based on Hyperledger Fabric to improve IoT device traceability and scalability. Islam et al. [5] protected industrial IoT data using differential privacy in a permissioned Blockchain.

To protect transaction confidentiality in terms of privacy and trust management, Malik et al. [6] proposed PrivChain, leveraging zero-knowledge proofs. For Blockchain supply chains, Putra et al. [7] developed a decentralized trust and reputation management model—DeTRM. Though this framework enhances privacy and data credibility, the automation features, such as IoT-based event validation or conditional payments, are not incorporated. Blockchain has been widely employed in supply chain management to strengthen traceability and authenticity. Kamble et al. [8] and Park et al. [9] explored the operational aspects of Blockchain for sustainable supply chain integration, but the performance at the implementation level is not taken into consideration. Hübschke et al. [10] offered a comprehensive review of Blockchain-based logistics networks and identified scalability and interoperability as ongoing challenges.

Most existing solutions focus on privacy, traceability, or trust management aspects of the integration of Blockchain and IoT individually. A comprehensive permissioned framework comprising IoT-driven conditional verification, NFT-based asset representation, and smart-contract-controlled escrow within a permissioned Blockchain infrastructure is yet to be developed and explored. The proposed framework deploys an integrated model that attains automation, verifiable asset transfer, along with a low latency of 0.0003 s, and monitoring performance metrics. Table 1 depicts the contribution of existing work in supply chain management aspects, such as transparency, traceability, asset tokenization, and real-time IoT data validation simultaneously. The proposed system, deployed on a permissioned Hyperledger Fabric environment, integrates all these functionalities, ensuring privacy, verifiability, and automation without the additional overhead to be borne in public networks. The incorporation of Prometheus–Grafana for continuous monitoring distinguishes this approach from the existing Blockchain-based models that rarely include the provision of monitoring.

This paper continues to present the design and analysis of the proposed framework. Section 2 is a description of the system architecture, asset and escrow models, and the Hyperledger Fabric implementation environment. Also, we discuss specifying the smart contract logic and condition-based validation workflow, as well as the implementation steps that are facilitated by event-based triggers. Section 3 provides the functional evaluation outcomes, such as the validation of workflow, robustness checks, and monitoring insights gathered in Prometheus–Grafana. We explain the relative standing of the framework in comparison to the existing approaches. Lastly, Section 4 is a conclusion of the work and an indication of the future directions of further deployment to bigger organization settings.

## 2. Materials and Methods

### 2.1. System Architecture

A transparent and secure smart automotive supply chain is created by integrating IoT devices with Hyperledger Fabric, a permissioned Blockchain network. The framework incorporates three primary stakeholders, namely the manufacturer, dealer, and end customer, who communicate over the Blockchain network, ensuring secure transactions and verifiable asset management. An NFT is used as a unique digital twin of the physical asset car. Furthermore, IoT sensors embedded in the car or the manufacturing units continuously capture the location, condition, and usage status. The data so collected is then recorded on the Blockchain for immutable traceability [11,12,13].

**Figure 1:**
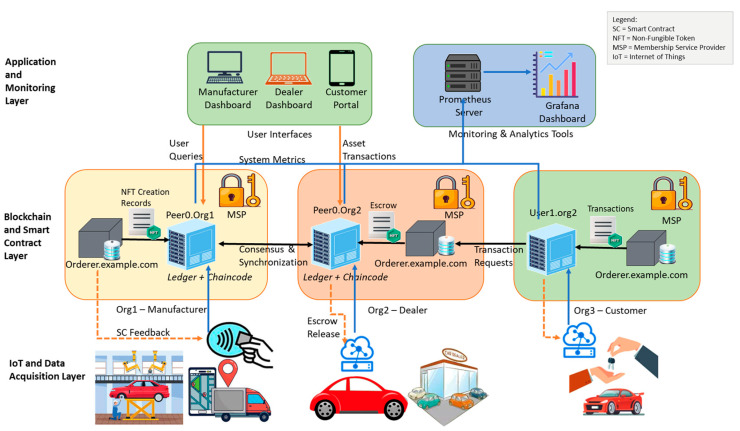
Architecture of the DeFiTrustChain.

The architecture of the proposed framework, as shown in Figure 1, consists of three major layers:

- *IoT and Data Acquisition Layer*: This will gather real-time information on sensors, RFID tags, and edge devices about the process of manufacturing and deliveries of cars [14,15].
- *Blockchain and Smart Contract Layer*: This layer is built on Hyperledger Fabric and is therefore responsible for transaction validation, the tokenization of assets and escrow-based payments, and transfer of ownership by use of chain code logic.
- *Application and Monitoring Layer*: This offers stakeholder user interfaces and dashboards. Performance metrics, such as network resource usage, are obtained through integration of Prometheus and Grafana components in this layer.

#### 2.1.1. NFT-Based Asset Representation

The proposed system utilizes Non-Fungible Tokens (NFTs), each of which contains the primary identity data of a vehicle, including the Vehicle Identification Number (VIN), as well as the manufacturer’s ID, production information, and ownership history. These NFTs are stored on the Blockchain register to ensure immutability and verifiable provenance [14,15,16]. Upon a vehicle being produced, the NFT smart contract on Hyperledger Fabric is triggered to generate one of the exclusive digital tokens (tokenID), which are attached to the car. The contract confirms the absence of a previous token on the same VIN, and the metadata of the given asset is logged together with the credentials of the manufacturer and the time when the ledger was distributed. After the record is created successfully, an event (CarNFTCreated) is emitted to confirm registration and launch other processes, including the processing of escrows and the transfer of ownership. The pseudocode of this NFT Creation Process is given in Algorithm 1 below, and it describes how a manufacturer can transform a real-life car into its inseparable digital version in the Blockchain (see Table 2).
**Algorithm 1.** NFT Creation Process**Input:** Asset details A, Owner O, SmartContract C **Output:** Unique NFT tokenID1: function CREATE_NFT(A, O) 2: Verify authenticity(A) 3: metadata ← generateMetadata(A) 4: tokenID ← hash(metadata || timestamp) 5: C.mint(tokenID, O, metadata) 6: Record transaction(tokenID, O) 7: return tokenID

#### 2.1.2. Escrow-Based Conditional Payments

In solving the issue of lack of trust amongst players in the supply chain, an escrow mechanism is applied in the Blockchain network. Payment transfers are automatically executed through smart contracts when predefined conditions are met. An example is where the dealer obtains a confirmed vehicle with the company, and the amount is automatically discharged from the escrow. In the same manner, the payment of the end customers is safely stored until the actual delivery of the cars is confirmed using the IoT sensors. This process helps minimize human intervention and transaction disputes while ensuring fairness and accountability among all parties (Algorithm 2).
**Algorithm 2:** Escrow Contract Initialization**Input:** Buyer B, Seller S, tokenID, price P, condition Cn **Output:** Transfer status 1: function ESCROW_TRANSFER (B, S, tokenID, P, Cn) 2: B → deposit(P) into escrowAccount 3: if verify(Cn) = TRUE then 4: transferToken(tokenID, S → B) 5:    releaseFunds(escrowAccount → S) 6: else 7:     refund(B) 8:     record transaction status 9:     return success/failure

#### 2.1.3. Data Flow and Transaction Process

The operational flow of the system is as described in the following steps:

- Registration and Enrollment: The members of every organization (manufacturer, dealer, customer) are registered in the Fabric CA and receive cryptographic identities.
- Asset Tokenization: The manufacturer creates a new NFT for every new car through a chain code function and stores it on the Blockchain.
- Escrow Initialization: When a dealer is willing to buy a car, an initial contract creates an escrow wallet, in which the money is stored.
- Check through IoT Data: IoT sensors ensure the delivery and car condition in real time and update Blockchain records. In Delivery Verification via IoT and Conditional Payment Release, presented as Algorithm 3, the pseudocode explains how verification is carried out and how payment is released after meeting the required conditions.
- Conditional Payment Release: When verification is successfully completed, the escrow smart contract will automatically release payment and transfer ownership of the NFT to the buyer. Algorithm 4 Administrative Dispute Resolution solves the problem in case the escrow status is discovered to be disputed.
- Monitoring and Analysis: Prometheus gathers metric performance data of peer and orderer nodes, and Grafana displays network health and throughput [17,18].

**Algorithm 3:** Delivery Verification via IoT and Conditional Payment Release**Input:** EscrowID, IoTData **Output:** Updated NFT ownership and escrow status 1. Escrow ← Ledger.Get(EscrowID) 2. if Escrow.Status ≠ “Locked” → return “Invalid escrow” 3. Verified ← CheckIoTConditions(IoTData, Escrow.Conditions) 4. if Verified = TRUE then 5.  if TransferFunds(Escrow.Buyer, Escrow.Seller, Escrow.Amount) = “Success” then 6.     NFT ← Ledger.Get(Escrow.TokenID) 7.     NFT.Owner ← Escrow.Buyer; NFT.Status ← “Delivered” 8.     NFT.History.Append(Escrow.Buyer, CurrentTime()) 9.     Escrow.Status ← “Released” 10.   Ledger.Update(NFT, Escrow) 11.   EmitEvent(“EscrowReleased”, EscrowID) 12.   return “Ownership transferred” 13.   else return “Payment failed” 14. else Escrow.Status ← “Disputed”; Ledger.Put(Escrow.ID, Escrow) 15. EmitEvent(“EscrowDisputed”, EscrowID); return “Verification failed”

**Algorithm 4:** Administrative Dispute Resolution**Input:** EscrowID, Decision **Output:** Updated Escrow.Status1. Escrow ← Ledger.Get(EscrowID) 2. if Escrow.Status ≠ “Disputed” → return “Invalid state” 3. if Decision = “Release” then Escrow.Status ← “ManuallyReleased” 4. else if Decision = “Refund” then Escrow.Status ← “Refunded” 5. else if Decision = “Cancel” then Escrow.Status ← “Cancelled” 6. else return “Invalid decision” 7. Escrow.UpdatedAt ← CurrentTime() 8. Ledger.Put(Escrow.ID, Escrow) 9. EmitEvent(“EscrowResolved”, EscrowID, Decision) 10. return “Resolution successful”

The administrative dispute resolution mechanism indicates a governance-based design preference that most permissioned enterprise networks usually take, where certain authorities handle exceptional cases.

#### 2.1.4. Monitoring and Performance Evaluation

To measure the effectiveness of the suggested framework, Prometheus and Grafana are combined to monitor the real-time system. Measures like block generation speeds, latency in transactions, peer response time, and resource utilization are constantly measured. The resulting metrics are visualized via Grafana dashboards that enable the researchers to evaluate the system scalability and performance when the workloads vary. This architecture will make the Blockchain network efficient and stable despite the increase in the number of IoT transactions.

### 2.2. Proposed Methodology

The proposed approach, DeFiTrustChain, is a combination of asset management based on Blockchain, data acquisition using IoT, and automation using escrow to create an open and reliable automotive supply chain environment. Hyperledger Fabric is at the core of data safety and privacy during communication between stakeholders, and smart contracts and NFTs guarantee asset verification and the impossibility of manipulation and falsification of payment terms [19]. Constant assessment and optimization of the system performance is also contributed toward by the integration of monitoring tools. The system architecture provided in Figure 1 is divided into three layers, which are under the IoT and data acquisition layer, the Blockchain and smart contract layer, and the application and monitoring layer. The IoT layer receives current vehicle data in real time, that then processed and stored on a Blockchain network controlled by Hyperledger Fabric [20,21]. Smart contracts also automate the process of managing assets and making payment transactions, and the application layer enables visualization and monitoring performance using Prometheus and Grafana.

**Figure 2:**
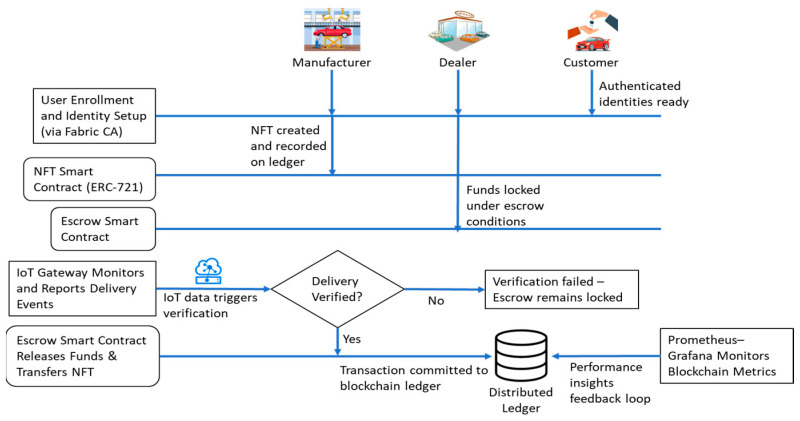
Workflow of the proposed Blockchain- and IoT-enabled methodology.

Figure 2 illustrates the sequential processes of the proposed system, starting with the stakeholder registration with Fabric CA, the creation of assets based on NFT, escrow payment initiation, delivery verification by IoT, and automatic release of payments with smart contracts [22,23,24,25,26]. The final step is the update of the ledger and constant monitoring of the network performance with the help of Prometheus and Grafana.

### 2.3. Implementation Environment

The suggested framework is deployed to the Hyperledger Fabric v2.x platform in order to facilitate secure and permissioned communications between network participants. The test network is composed of three organizations (Org1 (Manufacturer), Org2 (Dealer), and the Orderer Organization) with one peer node and a certificate authority (CA) each. The cryptographic materials are created using Fabric CA rather than cryptogen, so as to offer dynamic identity management as registration and enrollment take place. The deployed network setup is a focused experimental setup that will enable the appraisal of the practical operations of the proposed structure. It consists of NFT-based identity, escrow enforcement, and IoT-based verification. This setup eliminates the quasi-experimental impact of mass distributed implementation, and it is possible to test the architecture, transaction logic, and contract interactions. Even larger multi-organization supply chains, supported by Hyperledger Fabric, may still utilize the complete concept of system flow and design, as demonstrated in this implementation.

i. **Configuration for implementation in WSL-based Ubuntu is as follows:**
  - Intel Core i7 Processor (2.6 GHz, 12 Cores).
  - 16 GB RAM
  - Docker Engine: 27.x and Docker Compose: 2.x.
  - chaincode: GoLang v1.21 and Node.js v18.x client SDK
  - Prometheus v2.52 and Grafana v10 to monitor performance.
ii. **Blockchain Network Implementation:** The implementation was deployed on a two-peer Hyperledger Fabric 2.x Blockchain on Raft ordering service. Chaincode was run by each peer in external service mode, and the endorsement policy was AND(Org1.member, Org2.member). The block size was set to 10 transactions or 1 s, whichever came first.
iii. **Chaincode Implementation:** The implementation of the smart contract was carried out in Go and included registration of NFTs, state transitions, and escrow automation. In the contract, the deterministic state transitions are enforced by three major functions: FundEscrow, ReleaseEscrow, and CancelEscrow, each of which validates the current state (CREATED, FUNDED, RELEASED) before authorizing modifications.
iv. **IoT Interaction Logic:** IoT triggers were modeled through simulations through client applications, giving structured input to the chaincode in the form of JSON. Before escrow releases, the chaincode either checks conditions provided by the IoT (e.g., delivery confirmation flag, sensor-based checkpoints) or does not.
v. **Client Application Workflow:** All the transactions were placed through Fabric Gateway SDK in Go. The workflow consisted of the following actions: asset registration → escrow creation → IoT-triggered validation → escrow release.

All the smart contracts (chaincodes) were implemented in Go, with the main functions to create NFTs, initialize the escrow contracts, verify the IoT-based event, and transfer the ownership conditionally, as outlined in Algorithms 1–4. The orderer node is finalized the block by the consensus of both peer organizations before executing and endorsing each transaction, therefore ensuring consensus and immutability. The IoT input in this paper is represented by application-supplied event messages that form the simulation of sensor-derived conditions. This approach enables testing of the blockchain interaction workflow without relying on a physical IoT device instance.

#### 2.3.1. Network Deployment and Components

The implementation procedure was the standard Hyperledger Fabric process:

- Network Generation: The artifacts of channel configuration (genesis block and channel.tx) were generated with the help of configtxgen.
- CA-Based Identity Setup: Org1, Org2, and the OrdererOrg were started with separate CA servers, and peer, admin, and user identities could be registered using fabric-ca-client.
- Creation and Joining of a Channel: The orderer created the channel mychannel, and the peers of both organizations joined it.
- Chaincode Lifecycle: Both organizations installed, approved, and committed nftescrow.tar.gz.
- Application Invocation: NFT creation, escrow initiation, and events of verification were all represented using client SDK scripts to perform transactions.
- Performance Monitoring: Prometheus was used to gather real-time metrics of Fabric via the peer and orderer endpoints, whereas Grafana was used to display throughput, latency, and resource utilization.

#### 2.3.2. Experimental Parameters

To test system behavior, several test cases were run with controlled workloads:

- **Scenario 1:** One NFT transfer and creation between Org1 and Org2.
- **Scenario 2:** Several simultaneous escrow agreements between the dealer and the manufacturer.
- **Scenario 3:** Simulation of verification events of the IoT, which trigger a conditional ownership transfer.
- **Scenario 4:** A stress test including 100 sequential transactions to monitor block generation time.

The metrics measured included the following:

- Transaction latency (s): It is the time lag between the proposal of the transaction and its commitment.
- Throughput (TPS): The count of effective transactions per second.
- Resource usage percentage: The CPU and memory usage of peer and orderer containers.
- Escrow success rate (%): A ratio of successful conditional transactions to the number of transactions initiated.

Implementation-level artifacts, including chaincode interface specifications, deployment steps, and configuration templates.

## 3. Results

The Hyperledger Fabric v2.x was used to implement the proposed Blockchain-IoT framework, and Prometheus and Grafana were used to monitor it. Two peer organizations (Org1 and Org2) and one ordered node were configured, both of which were administered through Fabric CA. Blockchain height, transaction throughput, latency, and resource usage metrics were gathered when creating NFTs, transferring the escrow, and transferring ownership. Besides the performance assessment, a qualitative comparison was also made to outline the functional differences between the suggested framework and the current Blockchain-based supply chain systems, i.e., TruCert (2022) [1], IoTChain (2021) [6], and OriginTrail (2023) [7]. The analysis was based on such areas as authentication, automation, transparency, and integration with IoT. The suggested network based on the Hyperledger Fabric can be characterized as having better identity control through Fabric CA, complete automation of smart contracts through NFT, escrow, and verification modules, and the ability to integrate IoT data in real-time.

### 3.1. Quantitative Analysis

**Figure 3:**
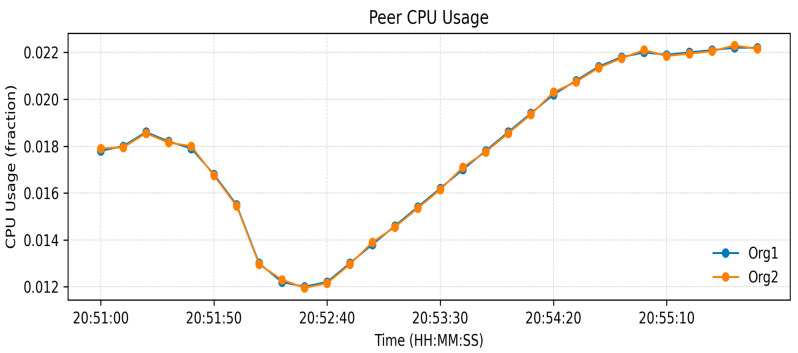
CPU utilization by manufacturer and dealer.

**Figure 4:**
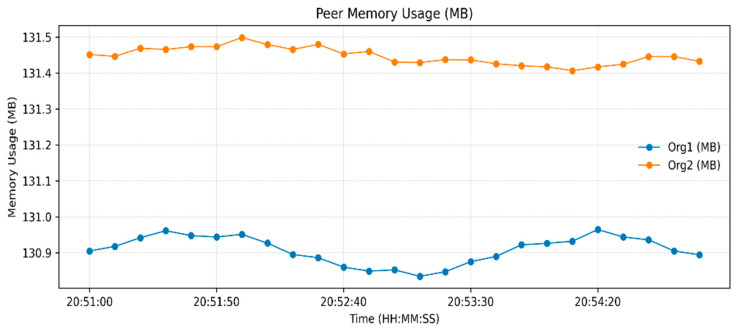
Memory utilization by manufacturer and dealer.

**Figure 5:**
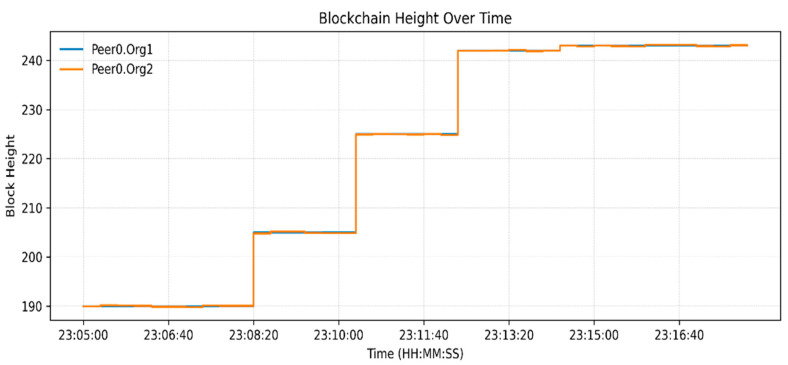
Block height for orderer, peer Org1 and peer Org2.

**Figure 6:**
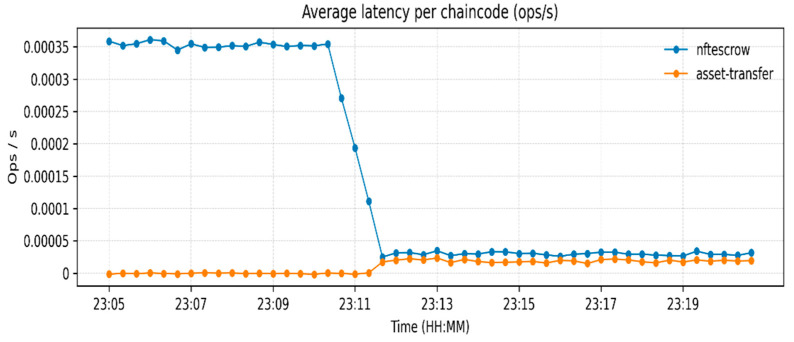
Latency for assettransfer and nftescrow chaincodes.

**Figure 7:**
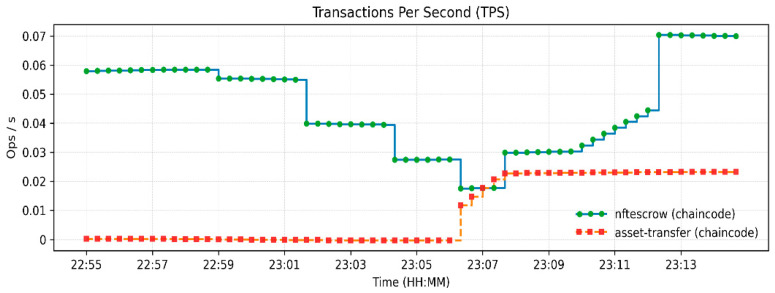
Transactions completed per second.

The measurements of resource utilization found in Figure 3, Figure 4 and Figure 5 relate to a low-load functional verification step of the proposed framework. Current solutions, even though they are effective at storing data and throughput, use partial or off-chain operations, which restricts traceability and trust. Table 3 indicates an increase in Blockchain height from 190 to 245 blocks and the peer ledgers were in tandem. Monitoring the operations of the orderer, 14 simultaneous requests were handled, which confirms the stable broadcast activities. CPU usage remained under 2.4% (Figure 3), memory under 131 MB, and block generation at 1 block/s (Figure 4 and Figure 5), respectively, which shows lightweight resource requirements and stable internal processes. Chaincode Latency remained at or below 0.0003 s, and assettransfer and nftescrow contracts all stabilized with warm-up, which were uniformly executed (Figure 6). The observed throughput was 0.06 ops/s (approximately 3–4 transactions per minute), and all requests were successfully committed (Figure 7), confirming functional correctness. The given throughput is associated with a test environment carried out to conduct a functional check of the suggested workflows.

### 3.2. Robustness Validation of Escrow Contract Logic

Two cases of negative-path tests were implemented in the deployed Hyperledger Fabric network to justify the enforcement of proper and conflict-free state transactions by the escrow contract. In the former case, escrow entry (ESCROW300) was funded and disbursed successfully, with a subsequent invocation of FundEscrow being invoked. The chaincode denied this operation with the error escrow ESCROW300 not in CREATED state, and this shows that re-funding an already released escrow is denied. In the second scenario, a CancelEscrow request was sent with the escrow already in the RELEASED terminal state. The smart contract once again refused the operation, stating that it cannot cancel the escrow, ESCROW300, that had already been released. Table 4 demonstrates that these tests establish that the contract correctly rejects invalid or conflicting actions, is consistent in escrow states, and rejects twice-spent or reclaimed consumed escrow money, so a simple robustness checking mechanism of the contract is that the contract prohibits mishandling or historic mistakes. Transactions completed per second is shown Figure 7.

### 3.3. Comparative Analysis with Existing Research

The framework that is proposed is compared with other similar Blockchain-based solutions in supply chains, including TruCert, IoTChain, and OriginTrail, to put the attained results into perspective. Table 5 represents a Quantitative comparison with platform type, asset representation, automation, and performance. Latency and throughput values for existing frameworks are taken from published studies and represent their reported performance under respective network configurations. The proposed framework’s metrics reflect execution on a two-peer Hyperledger Fabric prototype. Table 6 presents a functional comparison that highlights differences in identity handling, automation, traceability granularity, IoT integration depth, and monitoring visibility.

Existing works, namely TruCert and OriginTrail, operate on public or hybrid networks with a higher latency and limited privacy guarantees. The proposed permissioned Fabric design ensures deterministic transaction validation and controlled data visibility suitable for supply chain environments. Contrary to the IoTChain that authenticates only the states of devices, the current framework presents NFT-based representations of assets and conditional payments based on escrows, which allow end-to-end automation and traceability. The implementation uses simulated JSON-based inputs to model IoT event triggers, allowing systematic testing of smart-contract logic, workflow execution, and escrow conditions in a controlled environment. This design option enables the framework to be application-layer behavior-oriented and Blockchain-mediated interaction-oriented. Although the current two-peer deployment results in low throughput in a single-host setting, the evaluation effectively demonstrates the correctness of the end-to-end workflow. It is also distinguished by the addition of Prometheus–Grafana monitoring, which allows the obtention of continuous on-chain observability, an aspect rarely explored in existing Blockchain–IoT studies.

The comparative outcomes prove that the suggested system provides a differentiated combination of privacy preservation, automated condition-driven execution, and asset traceability within a permissioned Blockchain environment. The existing approaches address these aspects individually. The present framework integrates NFT-based asset representation with IoT-triggered verification and escrow-controlled settlement to support trustworthy digital transactions. These capabilities position the design as a promising foundation for domains that require secure and auditable asset exchanges.

## 4. Conclusions

The work introduces a Blockchain empowered, IoT-aided framework integrating Hyperledger Fabric smart contracts, NFT-enabled representation of assets, condition-grounded validation, and escrow-controlled settlement to facilitate traceability and automated decision implementation in automotive supply chains. The implementation shows perfect end-to-end behavior involving NFT-based asset registration, IoT-based verification, and conditional state transitions, and monitoring the system behavior with Prometheus–Grafana monitoring to enhance the transparency. The evaluation concentrates on validating functional interactions between Blockchain components, IoT data inputs, and conditional payment logic, rather than optimization of throughput in large-scale deployments. This unified workflow addresses gaps in prior approaches, which typically handle only isolated elements of automation within the supply chain. The proposed framework provides a practical foundation for secure and accountable asset exchange in multi-party automotive ecosystems by enabling condition-based automation and privacy-controlled collaboration among stakeholders.

Future work will expand the deployment to multiple organizations and peers to evaluate the behavior of the framework in larger consortium settings under higher transactional loads. The extension will allow a fuller assessment of scalability, endorsement distribution, and inter-organizational workflow performance. Future extensions on vulnerability analysis research, such as adversarial attack models and testing with regard to possible vulnerabilities in escrow execution, identity management, and transaction processes, can be incorporated. The incorporation of a real-time stream of IoT sensors and physical-layer dynamics is a natural continuation of the proposed work and will be investigated in the future. Additionally, multi-party or consensus-based dispute resolution systems can be utilized to further decentralize decision-making in escrow resolution.

## Figures and Tables

**Table 1:** Comparative analysis of existing Blockchain–IoT frameworks.

Framework	Platform	Asset Tokenization	IoT Integration	Conditional Payment/Escrow	Privacy Preservation	Permissioned Network	Performance Reported	Monitoring Support
TruCert [1]	Ethereum (public)	✗	✗	✗	✓	✗	~3.1 s latency	✗
Food Safety Chain [3]	Ethereum	✗	✓	✗	✗	✗	~2.8 s latency	✗
IoTChain	Private chain	✗	✓	✗	✓	✓	~1.2 s latency	✗
PrivChain [6]	Hyperledger + ZKP	✗	✗	✗	✓✓	✓	~1.5 s latency	✗
DeTRM [7]	Custom Blockchain	✗	✓	✗	✓	✓	Not reported	✗
OriginTrail	Polkadot Layer-2	✓ (graph assets)	✓	✗	✗	✗	~0.8 s latency	✗
Fabric–Tea Chain [2]	Hyperledger Fabric	✗	✓	✗	✗	✓	~0.6 s latency	✗
Sustainable Supply Chain [8,10]	Ethereum	✗	✗	✗	✓	✗	~3.5 s latency	✗
DeFiTrustChain	Hyperledger Fabric (permissioned)	✓ (NFTs)	✓✓ (real-time IoT)	✓✓ (escrow automation)	✓ (CA-based)	✓✓	0.0003 s latency, 0.06 TPS	✓ (Prometheus–Grafana)

(✓✓ = strong support, ✓ = partial support, ✗ = not supported).

**Table 2:** Notation used.

Symbol/Function	Meaning
Ledger.Put(key, value)	Write data to the Blockchain ledger
Ledger.Get(key)	Retrieve data from the ledger
EmitEvent(name, data)	Trigger Blockchain event for monitoring
CheckIoTConditions(data, cond)	Verify IoT payload against escrow conditions
TransferFunds(buyer, seller, amount)	Execute payment transfer (tokenized or off-chain)
CurrentTime()	Returns system timestamp
TxID	Unique transaction identifier

**Table 3:** Recorded performance metrics from Prometheus–Grafana monitoring.

Metric	Observed Value/Range	Remarks
Blockchain Height	190 → 245 blocks	≈55 new blocks generated during execution.
Peer Height (Org1/Org2)	20 each	Ledgers synchronized across peers.
Orderer Active Requests	14	Stable broadcast/deliver operations.
Chaincode Access Latency (GET/PUT)	0.0001–0.0003 s	Sub-millisecond state DB response time.
Avg. Latency—assettransfer	0.00035 → 0.0001 ops/s	Stabilized after initialization.
Avg. Latency—nftescrow	≈0.00015 ops/s	Consistent under escrow logic.
Requests Completed	0.24–0.26 ops/s	All transactions successfully committed.
Transaction Throughput (TPS)	0.06 ops/s	≈3–4 transactions/minute.
Peer CPU Usage	1–2.4%	Minimal computational overhead.
Peer Memory Usage	68–131 MB	Lightweight container operation.
Orderer Throughput	≈1 block/s	Continuous block production maintained.

**Table 4:** Negative-path escrow validation tests.

Test Case	Invalid Operation Attempted	Chaincode Response	Outcome
Re-funding after release	FundEscrow invoked on already released ESCROW300	“Escrow ESCROW300 not in CREATED state”	Rejected—prevents reactivation
Cancelation after release	CancelEscrow invoked after escrow released	“Cannot cancel escrow ESCROW300 already released”	Rejected—enforces terminal state

**Table 5:** Quantitative evaluation with existing blockchain supply chain frameworks.

Framework	Platform	Core Feature	Asset Type	Automation	Avg. Latency	Throughput (TPS)	IoT Integration
TruCert (2022) [1]	Ethereum (public)	Product authenticity	Fungible token (ERC-20)	Smart-contract verification	~3.1 s	15–20	Limited (manual)
IoTChain (2021) [6]	Private chain (PoW)	IoT data integrity	Device IDs	Edge-level consensus	~1.2 s	25–30	Strong (edge nodes)
OriginTrail (2023) [7]	Polkadot + Layer-2	Data sharing	Graph objects	Off-chain sync	~0.8 s	40	Medium (API)
DeFiTrustChain	Hyperledger Fabric (permissioned)	NFT-based asset + escrow automation	Non-Fungible Token (NFT)	Smart-contract + IoT verification	0.0003 s	0.06 TPS (2-peer)	Full real-time IoT integration

**Table 6:** Qualitative comparison with existing Blockchain-based supply chain frameworks.

Framework	Identity Management	Automation	Traceability	IoT Integration	Monitoring
TruCert (2022) [1]	Public key-based	Basic token validation	Moderate	Manual verification	Limited
IoTChain (2021) [6]	Device-level keys	Edge consensus	Good	Strong (edge IoT)	Basic logs
OriginTrail (2023) [7]	API-based access	Off-chain sync	Medium	Partial	Absent
DeFiTrustChain	Fabric CA-based digital identity	Full NFT + Escrow + Verification automation	End-to-end immutable tracking	On-chain real-time IoT verification	Continuous Prometheus–Grafana monitoring

## Data Availability

The data used are from online resources, and simulated data were used for verification.

## Abbreviations

The following abbreviations are used in this manuscript:

NFT	Non-Fungible Token
CA	Certificate Authority
WSL	Windows Subsystem for Linux
TPS	Transactions per Second
